# Social Big Data as a Tool for Understanding and Predicting the Impact of Cannabis Legalization

**DOI:** 10.3389/fpubh.2019.00274

**Published:** 2019-10-04

**Authors:** Sean D. Young, Howard Padwa, Erin E. Bonar

**Affiliations:** ^1^University of California Institute for Prediction Technology, Department of Informatics, University of California, Irvine, Irvine, CA, United States; ^2^Department of Emergency Medicine, University of California, Irvine, Irvine, CA, United States; ^3^Integrated Substance Abuse Program, University of California, Los Angeles, Los Angeles, CA, United States; ^4^Department of Psychiatry, Addiction Center, University of Michigan, Ann Arbor, MI, United States

**Keywords:** cannabis, social media, big data, drug abuse, prevention

## Introduction

After alcohol and tobacco, cannabis is the most commonly used substance in the United States (U.S.), with 8.9% of individuals ages 12 and older reporting past-month use (1). Cannabis policy is currently undergoing a historic change in the United States. Though cannabis is illegal under federal law, 29 states and the District of Columbia (D.C.) now allow medical use, and since 2012, eight states and D.C. have passed laws permitting non-medical use (2).

In California, voters approved non-medical use of cannabis through a ballot initiative in November 2016. With a population of nearly 40 million, California has a larger population than the other states with legalized non-medical use combined (3). Because of its size, California may offer an unprecedented opportunity to examine the consequences of legalization, and to glean lessons about legalization's impacts that will be essential as policymakers and regulators in other states (and potentially at the federal level) consider cannabis legalization in the future. In this article, we highlight our viewpoint on some of the major lessons about cannabis legalization that can be learned in California, and how big data can be used to generate knowledge about cannabis legalization's impact on public health and safety.

## Evaluating Legalization's Impact

We believe there are reasons to be both optimistic and concerned about legalization's impact. First, cannabis legalization holds promise as a way to reduce the criminal justice-related costs of prohibition (e.g., incarceration for cannabis-related offenses, enforcement costs), decrease the cannabis black market, facilitate the regulation of cannabis products for purity and safety, generate tax revenues (4), and increase access for individuals who use cannabis without experiencing significant negative consequences or impaired functioning. However, legalization may increase cannabis use, putting more individuals at risk for cannabis use disorders and other mental health, medical, and psychosocial problems associated with the drug (5). Legalization may increase rates of cannabis consumption in ways that increase risk to public health and safety, such as driving while under the influence (6), and overconsumption of edibles or concentrates leading to psychiatric distress that may require emergency care (7, 8). These risks are of particular concern since the potency of cannabis—as measured by THC levels—has been consistently rising over the past two decades (9).

Given these potential benefits and harms, there has been significant interest in analyzing survey, health system, and administrative data from the first states to legalize non-medical use in order to evaluate legalization's impact. A major focus of this research has been the impact of legalization on rates of cannabis use, the frequency of use, and use by individuals who are at particularly high risk for health or psychosocial problems because of cannabis (10–15). Legalization's impact on negative outcomes related to increased use—such as cannabis-related hospital and emergency department visits, poison control center calls, motor vehicle crashes, and cannabis use disorder treatment episodes—has also received significant attention (11, 15–19). Shifts in cannabis markets, production, sales, and tax revenue are also being monitored (15, 17, 20). The potential benefits of legalization have garnered less attention, though quasi-experimental research suggests that cannabis legalization may be associated with reductions in opioid prescribing and opioid-related deaths (21, 22).

We believe this research is helping develop the knowledge base about critical health and policy questions related to cannabis legalization, but the data sources have limitations. There is a considerable time lag between policy implementation and being able to collect and analyze data on the impact of policy changes, making it difficult to fully understand legalization's consequences in a timely manner. Many measures of cannabis use on existing surveys only ask about prevalence or frequency of use, but do not collect information about amounts of cannabis consumed, the potency of cannabis being used, or what other substances (e.g., alcohol, tobacco) are co-ingested with cannabis (23). Available data typically rely on self-report, which may be imprecise because of difficulty measuring and reporting cannabis consumption in absence of accurate information about potency or “dose.” Information concerning legalization's adverse effects collected from hospitals, poison control centers, law enforcement, and substance use disorder treatment providers often have small sample sizes and short time frames, though these data will become more robust as they are collected in more places and over longer periods of time (24). Analyses of sales data are generating significant insights into how legalization is impacting the price of cannabis and the nature of products being sold, but it remains challenging to evaluate the impact these products have on the individuals purchasing and consuming them (23).

Another issue is that the datasets being used to inform analyses of legalization's impact are designed to measure and evaluate legalization's potential negative outcomes (e.g., increased use among at-risk individuals, hospitalizations, accidents), but are ill equipped to capture information concerning legalization's potential benefits. Though some research has examined the relationship between legalization and reductions in negative outcomes (21), existing datasets do not collect information about the benefits of legalization—such as increased access to a substance that many individuals find pleasurable and can use with little ill effects. To comprehensively evaluate legalization's impact such measures of its benefits will be essential.

Finally, existing methods of collecting data on cannabis trends— such as surveys, medical data, and interviews—are expensive, rely on cumbersome and time-consuming processes to secure funding, and require significant amounts of time and staff resources. With these traditional methods, researchers may not be able to identify the societal impact of cannabis policy changes for many years. It is imperative that researchers can begin to explore the impacts of legalization prospectively in order to learn about potential benefits and to address drawbacks of the policy and growing concerns before they become significant public health problems.

## How Big Data Can Enhance Our Understanding of Legalization's Impact

Methods to analyze “big data” might be leveraged to advance our understanding of cannabis legalization's impact. As big data have penetrated our daily existence, large amounts of medical, environmental, genomic, and public health data, paired with publicly available data from social media and search engines can provide critical information about public health problems. However, new methods are needed that are capable of collecting and analyzing these data. These approaches may be useful in surveillance efforts to help public health and policy researchers understand and predict the implications of changing trends in cannabis policies (25).

For example, one area of big data research that may be particularly helpful involves the study of “social big data,” such as data from social media, wearable devices, and online search data. One in four people worldwide are publically documenting their activities, intentions, moods, and social interactions on social sites each day (26). They are increasingly doing so, generating 400 million “tweets” per day on Twitter (27) and 4.75 billion content items posted each day on Facebook (28). Most of these platforms support user profiles, tagging, time-stamping, and/or geolocation capabilities, making key demographic and contextual information available for analysis. Many platforms also provide data on users' social network connections, allowing access to new and valuable information on how social groups influence attitudes, behaviors, and health (29). For example, HIV researchers have studied the content people display on their social networking profiles and used this information to inform HIV research (30–32). These studies have found that social media posts contain information about people health behaviors, such as their drug use and risk behaviors, and that these conversations can be mapped on a US map and used to help inform public health and surveillance efforts. Similarly, social media data regarding cannabis use has implications for interventions (33–36), such as identifying trends in cannabis use or abuse across counties or states, as well as identifying new ways that cannabis is being used as described in social media.

Much of the available social data exists in an unstructured format, such as free-text social media posts which would require researchers to read and analyze them (37–39). However, computer science/machine learning methods could be used to train machines to learn the patterns identified by human domain experts in detecting whether posts are related to cannabis or not. This process could lead to instant identification of millions of real-time social media posts about cannabis (40, 41). While sifting through these posts could take researchers years to complete, machines could do so within seconds.

Once posts have been identified as being related to cannabis or not, data could be tagged for location to triangulate location data (i.e., identifying potential hot spots) with actual cannabis outcomes data for use in models attempting to predict cannabis-related outcomes, such as trends in cannabis use, or motor vehicle crashes related to driving under the influence. For example, researchers at the University of California Institute for Prediction Technology (UCIPT) partnered with the California Highway Patrol (CHP) and the crowdsourced app, Waze, to study whether Waze data might predict reported crashes, before these events were reported to the CHP. Waze data on reported incidents were labeled as to whether they were car accidents or potential car accidents and merged with actual CHP data, then plotted on a map of California together. Machine learning models identified whether Waze reports of accidents were reported prior to CHP reported accidents. Results suggested that Waze reported accidents ~3 min faster than CHP reports (42). This provided a model for how social data might be used to monitor events in real-time so that public health officials and first responders could intervene faster than with current methods. Cannabis use researchers might apply these social big data methods similarly to gain an understanding of real-time cannabis-related outcomes before they are even reported.

Machine learning methods can be used not only for monitoring public health issues and trends, but also as a tool for prediction. For example, social media and online search data have been used to predict future outbreaks of syphilis, rates of heart disease, and many other public health events (43–45), which can then inform adjustments to policies or community-level interventions. These methods may be especially helpful for monitoring and predicting events in areas where limited data exist or where it is expensive and takes a long time to gain data on a topic that needs to be understood quickly, such as the implications of cannabis use. Similarly, cannabis-related data triangulated between social media and other mhealth sources (e.g., geolocation, analyses of movement, ambient noise), might be used to predict presence of cannabis use disorders or related co-occurring conditions (e.g., anxiety, major depression), perhaps identifying systems (e.g., school, healthcare), or communities for treatment resource allocation. In addition, applying social big data to the individual can inform personalized medicine approaches to delivering mhealth interventions to prevent further negative consequences which could be instantaneously initiated through social media. Social big data could also yield insights regarding the nature of non-hazardous cannabis use, perhaps by identifying features of social networks and individuals that can be leveraged to increase safer use of the drug among legal users. Furthermore, machine learning methods could potentially be utilized to analyze social media data concerning the potential benefits of legalization—such as pleasure resulting from enhanced access or increased use—that may be difficult to measure using other surveys and other existing data collection methods.

## Conclusion

We believe that big data modeling approaches can be extremely useful for researchers and policymakers attempting to learn about the implications of cannabis legalization. If applied in real time to a large population, such as that of California, big data-based modeling approaches could provide an additional tool learn about cannabis reforms' benefits and costs, and inform the development of evidence-based public health and public policy in the age of legalization.

## Author Contributions

SY conceived of the concept, wrote the section on big data, and reviewed final draft. HP and EB helped provide input to the concept, wrote the introduction and concluding sections, and reviewed manuscripts drafts.

### Conflict of Interest

SY has received consulting funds, which may have affected experiences and/or views related to this manuscript. The remaining authors declare that the research was conducted in the absence of any commercial or financial relationships that could be construed as a potential conflict of interest.
